# Molecular characterization and phylogenetic analyses of MetAP2 gene and protein of *Nosema bombycis* isolated from Guangdong, China

**DOI:** 10.3389/fvets.2024.1429169

**Published:** 2024-06-28

**Authors:** Izhar Hyder Qazi, Ting Yuan, Sijia Yang, Christiana Angel, Jiping Liu

**Affiliations:** ^1^Guangdong Provincial Key Lab of Agro-Animal Genomics and Molecular Breeding, College of Animal Science, South China Agricultural University, Guangzhou, China; ^2^Shaheed Benazir Bhutto University of Veterinary and Animal Sciences, Sakrand, Pakistan

**Keywords:** *Bombyx mori*, microsporidia, *Nosema*, pebrine, sericulture

## Abstract

**Background:**

Pebrine, caused by microsporidium *Nosema bombycis*, is a devastating disease that causes serious economic damages to the sericulture industry. Studies on development of therapeutic and diagnostic options for managing pebrine in silkworms are very limited. *Methionine aminopeptidase type 2* (*MetAP2*) of microsporidia is an essential gene for their survival and has been exploited as the cellular target of drugs such as fumagillin and its analogues in several microsporidia spp., including Nosema of honeybees.

**Methods:**

In the present study, using molecular and bioinformatics tools, we performed in-depth characterization and phylogenetic analyses of MetAP2 of *Nosema bombycis* isolated from Guangdong province of China.

**Results:**

The full length of *MetAP2* gene sequence of *Nosema bombycis* (Guangdong isolate) was found to be 1278 base pairs (bp), including an open reading frame of 1,077 bp, encoding a total of 358 amino acids. The bioinformatics analyses predicted the presence of typical alpha-helix structural elements, and absence of transmembrane domains and signal peptides. Additionally, other characteristics of a stable protein were also predicted. The homology-based 3D models of MetAP2 of *Nosema bombycis* (Guangdong isolate) with high accuracy and reliability were developed. The MetAP2 protein was expressed and purified. The observed molecular weight of MetAP2 protein was found to be ~43–45 kDa. The phylogenetic analyses showed that MetAP2 gene and amino acids sequences of *Nosema bombycis* (Guangdong isolate) shared a close evolutionary relationship with *Nosema* spp. of wild silkworms, but it was divergent from microsporidian spp. of other insects, *Aspergillus* spp., *Saccharomyces cerevisiae*, and higher animals including humans. These analyses indicated that the conservation and evolutionary relationships of MetAP2 are closely linked to the species relationships.

**Conclusion:**

This study provides solid foundational information that could be helpful in optimization and development of diagnostic and treatment options for managing the threat of *Nosema bombycis* infection in sericulture industry of China.

## Introduction

Sericulture has been practiced since millennia and today holds a cultural legacy integral to the ancient history of China (1). Sericulture has not only provided subsistence to local rural economies, but it has been practiced at mechanized and industrial levels in recent times, particularly in China. Although sericulture, as a low-capital, high yield industry, has now moved into the global market (2), its sustainable and healthy development is facing several challenges including economic losses due to poor management practices and inadequate disease control measures in mulberry and silkworm production systems (3, 4).

The domesticated *Bombyx mori* L., the only truly domesticated insect species (5), is largely reared by silk producers to produce high quality silk threads of commercial importance. However, since many decades, silkworm production has been affected by many pathogens (6) causing serious economic damages to the industry.

Microsporidia are a fascinating and hyper-diverse group of obligate spore-forming intracellular parasites infecting a wide range of vertebrate and invertebrate hosts, including human and virtually all major animal taxa in all global biomes (7–10). To date, over 1,700 microsporidian species belonging to over 200 genera have been identified (9, 10). Many of these microsporidian parasites have been reported to infect several agriculturally important animal species including honeybees, shrimps, fish, and silkworms (11). However, despite their widespread occurrence and distribution, our understanding of their complex biology, pathogenic mechanisms, diagnosis, and treatment remains limited. The good news is that these areas have emerged as hot topics of research in recent times (12–16).

In 1857, microsporidium *Nosema bombycis* was identified as a causal pathogen of pebrine, a destructive disease of silkworms (17). *Nosema bombycis* has the ability to infect silkworms through fecal-oral (horizontal) and transovarial (vertical) routes (11), the latter causing a significant economic threat to sericulture production systems in major sericulture-intensive countries. At present, the precise mechanistic basis of the transovarial transmission and how *Nosema bombycis* parasite penetrates the host barrier system and enter into the oocytes remain poorly understood (11). However, recently it was shown that *Nosema bombycis* initially infects the follicular and nurse cells of the silkworm ovariole sheath, ultimately getting entry into the oocytes. Additionally, *Bombyx mori* vitellogenin and specific spore wall proteins of *Nosema bombycis* were shown to be key factors involved in the transovarial transmission (11).

When infected with *Nosema bombycis*, the silkworm larvae exhibit signs of delayed growth, molting problems, pustular milky white patches attached to the silk glands, and black spots on the entire body, eventually leading to death (18–21). Given that the obvious symptoms appear late in the infection, asymptomatic and mild infections are extremely deceptive, making the prevention, diagnosis and treatment of pebrine very challenging. Due to these reasons, pebrine remains the only disease with a mandatory quarantine (22) in the sericulture industry.

Previously, efforts were made to identify possible therapeutic targets and to develop treatment options to cure microsporidia infections, unfortunately results have ranged from disappointing to promising so far (21). We now know that methionine aminopeptidase type 2 (*MetAP2*) of microsporidia is an essential gene for their survival and has been exploited as the cellular target of drugs such as fumagillin, ovalicin, and TNP-470 (23). Fumagillin, the only approved veterinary drug for the treatment of *Nosema* infections in honey bees (21), can irreversibly inhibit *MetAP2* of microsporidia, blocking the essential enzymes and interfering the protein homeostasis necessary for pathogen survival (24, 25). However, it has also been shown that, despite its broader antimicrosporidial activity, fumagillin was not found to be effective against all microsporidia spp. (21, 24).

Given that the reports on therapeutic effects of drugs for treating *Nosema bombycis* infection in silkworms are very limited, it would be a timely effort to identify and characterize select genes that could be utilized as potential targets for specifically diagnosing and treating *Nosema bombycis* infection in silkworms [e.g., as argued in (26)]. Therefore, in the present study, building on our pioneering work (Qazi and Liu, 2024; unpublished results) on *de novo* transcriptome sequencing of *Nosema bombycis* (Guangdong isolate), we performed an in-depth study on identification, expression, and characterization of MetAP2 gene and protein using molecular and bioinformatics tools. Meanwhile, *MetAP2* gene of *Nosema antheraeae*, a microsporidium infecting the Chinese oak (Tussah) silkworms (27), was also sequenced and annotated for phylogenetic comparison. This study provides solid foundational information based on the local isolate of *Nosema bombycis*, and will pave a way for optimization and development of diagnostic and treatment options for managing the threat of *Nosema bombycis* infection in sericulture industry of China.

## Materials and methods

### Ethics statement

This work was conducted in accordance with the institutional ethical guidelines of South China Agricultural University, and other relevant International guidelines. No specific approval was needed, as currently research on insects including the silkworms does not require ethical approvals (28).

### Collection and purification of parasites and DNA extraction

*Nosema bombycis* spores were propagated and purified from silkworms (*Bombyx mori*) maintained in our laboratory as described previously (29). The pure suspension of *Nosema bombycis* spores was prepared at a concentration of 1 × 10^^8^ spores/mL. This suspension was uniformly spread on the back of mulberry leaves and fed to the fourth-instar silkworms. The confirmation of *Nosema bombycis* infection was done by the light microscopic examination of the dead silkworms. The dead silkworms were placed in a tissue homogenizer for homogenization, and the pure *Nosema bombycis* were recovered. Briefly, the homogenate was filtered through four layers of gauze and one layer of absorbent cotton. The filtrate was put into a 15 mL centrifuge tube and centrifuged at 500 r/min for 2 min to remove the silkworm body and sediment. Then, it was centrifuged at 3000 r/min for 15 min and the supernatant was discarded. The pellet was resuspended in ddH_2_O and centrifugation was continued three times until a clear supernatant was obtained. Finally, a relatively pure white spore pellet was obtained and stored at 4°C for further use.

Meanwhile, other microsporidia, including *Nosema antheraeae* (Host: *Antheraea pernyi;* Oak Silkworm), *Endoreticulatus* sp. Zhejiang (Host: *Bombyx mori;* Mulberry Silkworm), and *Nosema pyraustae* GZ (Host: *Pyrausta nubilalis,* Hubern; Corn Borer) were utilized from our laboratory-maintained collection. The genomic DNA of these microsporidia was extracted using the DNeasy Mini Kit (Qiagen, Germany) following the manufacturer’s instructions.

### PCR amplification of *MetAP2* gene

*MetAP2* gene of *Nosema bombycis* was annotated using *Nosema bombycis* (Guangdong isolate) transcriptome (Qazi and Liu, 2024; unpublished data), the MicrosporidiaDB^1^ and the National Center for Biotechnology Information (NCBI) (30) databases. The primers were designed using Primer 5.0 software (31) as follows: MC-F (5’-ATGAGGCCTATTGTTTTATCAGAAG-3′)/MC-R (5’-TTAAAAATCATTCCTTTTGTAAGA-3′), with an amplification length of 1,077 bp. *MetAP2* genes of three other species of insect microsporidia were amplified. These included *Nosema antheraeae*, *Endoreticulatus* sp. Zhejiang, and *Nosema pyraustae* GZ.

For PCR validation of *MetAP2* gene, DNA of other common pathogenic microorganisms of silkworms including *Bacillus bombysepticus*, *Bacillus thuringiensis*, *Beauveria bassiana*, *Bombyx mori* cytoplasmic polyhedrosis virus (BmCPV) polyhedron, and *Bombyx mori* nuclear polyhedrosis virus (BmNPV) was used as template. The DNA of these microorganisms was extracted using the DNeasy Mini Kit (Qiagen, Germany) following the manufacturer’s instructions. Sterilized water was used as a negative control.

The PCR reaction system comprised 12.5 μL of 2 × Taq PCR Master Mix, 1 μL each of 10 μmol/L primers MC-F/R, 2 μL of 10 ng/μL DNA template, and sterilized ddH2O up to a total volume of 25 μL. Amplification conditions were as follows: pre-denaturation at 94°C for 5 min; denaturation at 94°C for 30 s, annealing at 50°C for 40 s, extension at 72°C for 1 min for 32 cycles; final extension at 72°C for 8 min, and storage at 4°C. 5 μL of PCR amplification products were subjected to 1.2% agarose gel electrophoresis for detection.

### Construction and sequence analysis of *MetAP2* gene recombinant plasmid

The PCR amplification product was subjected to agarose gel electrophoresis, and recovered using the TaKaRa MiniBEST DNA Gel Recovery Kit (Takara Bio Inc.) as per the manufacturer’s instructions. The recovered DNA fragment was ligated into the pMD™19-T vector and transformed into DH5α competent cells. The desired bacterial strains were selected, and plasmids were extracted according to the instructions of the TaKaRa MiniBEST Plasmid Purification Kit (Takara Bio Inc.). Following PCR verification using the primers MC-F/R, the samples were sent to Sangon Biotech (Shanghai) Co., Ltd. for sequencing using the dideoxy method. The cloned sequencing results were analyzed using MegAlign Pro sequence alignment software (version 17.6) of DNASTAR Lasergene for multiple sequence alignment to assess the similarity of *MetAP2* gene across species. The sequence of *Nosema bombycis MetAP2* gene generated in the present study was submitted to the NCBI database with accession number KX185053.1.

### Annotation and functional prediction of *Nosema bombycis* MetAP2

The ORF finder^2^ (32) was used to identify protein encoding sequence. The predicted MetAP2 protein sequence of *Nosema bombycis* was verified using the Basic Local Alignment Search Tool (BLAST) on NCBI^3^ (33).

The predicted *Nosema bombycis* MetAP2 protein sequence was subjected to conserved domain prediction using CD-search^4^ on NCBI (34–36). The physico-chemical properties, molecular weight, and isoelectric point of MetAP2 protein were predicted using ProtParam online software of EXPASY^5^ (37). Transmembrane regions of amino acids were predicted using the DeepTMHMM (v.1.0.24)^6^ (38). The presence of signal peptides in amino acid sequence was predicted using the SignalP 6.0 Server^7^ (39). The secondary structure of protein was predicted using the Multivariate Linear Regression Combination (MLRC) method of online prediction software Network Protein Sequence Analysis^8^ (40), and protein sub-localization was predicted using Protein Subcellular Localization Prediction Tool (PSORT II)^9^ (41) and DeepLoc-2.0^10^ software (42).

### Homology and phylogenetic analyses of MetAP2 of *Nosema bombycis*

*MetAP2* gene and protein sequences of *Nosema bombycis* were BLAST-searched to retrieve similar sequences of other species on NCBI. Meanwhile, MetAP2 sequences were also retrieved manually using the MicrosporidiaDB (see text footnote 1) database. After filtering, sequences with high similarity to the *Nosema bombycis* MetAP2 were downloaded for homology and phylogenetic analyses. MetAP2 gene and protein sequences from different species including humans, animals, *Bombyx mori*, and *Aspergillus* spp., as well as MetAP2 sequences of *Nosema antheraeae* obtained in the present study, were aligned using the Clustal W. method (43) using MegAlign Pro sequence alignment software (version 17.6) of DNASTAR Lasergene. The phylogenetic analysis (molecular evolutionary trees) was performed using the latest Randomized Axelerated Maximum Likelihood (RAxML; version 8.2.12) method (44), which is based on the rapid bootstrapping algorithm. The bootstrap value was set to 100 replicates. The homology comparisons (percent identity and divergence) of MetAP2 with other species were also carried out using MegAlign Pro sequence alignment software (version 17.6) of DNASTAR Lasergene.

### Homology modelling

Homology modelling of 3D protein structure of MetAP2 was performed using the SWISS-MODEL software^11^ (45). This method uses template search which is performed against the SWISS-MODEL template library (SMTL, last update: 2024-02-21, last included PDB release: 2024-02-16). Models are built based on the target-template alignment using ProMod3 (46, 47). The global and per-residue model quality was assessed using the QMEAN scoring function (46, 47). Briefly, the protein sequence of MetAP2 of *Nosema bombycis* generated in the present study was used as target sequence to develop its 3D homology model. The search retrieved overall 662 templates, out of which 50 templates with high similarity were auto selected for model development. The templates which were considered to be less suitable for modelling were auto removed and the top 50 filtered templates were retained. In this case, we selected two best matching templates with higher quality estimation scores. These included: (1) A0A3G6ILP9.1.A methionine aminopeptidase 2 AlphaFold DB model of A0A3G6ILP9_9MICR (*MetAP2* of *Nosema assamensis*); (2) 3 fm3.1.A (X-ray Crystal structure of *MetAP2* of *Encephalitozoon cuniculi*).

### Prokaryotic expression of *Nosema bombycis* MetAP2

#### Construction of expression vector

The Primer Premier 5.0 software was used to analyze the restriction sites of *MetAP2* gene and to design primers incorporating dual restriction sites NdeI/XhoI (Supplementary Figure S1). Primers Pmet-F (F: 5’-GACACCATATGTTAGAAGCGAGGCGTGCAGCTG-3′) and Pmet-R (5’-GTGTCCTCGAGCTATTAAAAATCATCTCCTTTTG-3′) were designed for PCR amplification of *Nosema bombycis* DNA, yielding a product of 871 bp in length. The PCR amplification system and program were the same as described in sub-section “PCR Amplification of *MetAP2* Gene.” The PCR products were recovered using TaKaRa MiniBEST DNA Gel Recovery Kit (Takara Bio Inc.).

#### Ligation of target gene and vector

The recovered target fragment and vector pET-28a were digested with NdeI and XhoI, followed by ligation using T4 ligase. The transformation of the ligation products and extraction of recombinant plasmids were the same as detailed in sub-section “Construction and Sequencing Analysis of *MetAP2* Gene Recombinant Plasmid.” The PCR-verified positive bacterial culture was incubated at 37°C with shaking overnight for plasmid extraction, followed by restriction digestion with NdeI and XhoI. The positive cultures were sent to Sangon Biotech (Shanghai) Co., Ltd. for sequencing. The correctly sequenced recombinant plasmid was named pET-28A–met.

#### Transformation into *Rosetta* (DE3) strain

One μl of pET-28A–met was added to *Rosetta* (DE3) strain suspension and subjected to heat shock at 42°C for 90 s. After two min of rest on ice, the suspension was spread on plates (34 μg/mL chloramphenicol and 30 μg/mL kanamycin) and incubated overnight at 37°C. After transformation, the colonies were picked up aseptically for PCR verification.

#### Prokaryotic expression

Colonies containing pET-28A–met were picked and cultured in 2.5 mL LB medium (containing 34 μg/mL chloramphenicol and 30 μg/mL kanamycin) at 37°C and 220 rpm for approximately three h. When the OD value reached about 0.6, IPTG was added to a final concentration of 0.5 mM, and incubation was continued at 20°C overnight with shaking at 220 rpm. The cell pellets were collected by centrifugation, with non-IPTG-induced samples serving as the negative controls. The collected bacteria were resuspended in lysis buffer (1 × PBS, pH 7.4). The cell lysis was achieved through ultrasonication in an ice bath at 400 W power for 20 min. The suspension was centrifuged at 12000 rpm at 4°C for 20 min, and the supernatant was collected.

A five mL Ni-IDA column was equilibrated with 10 column volumes of binding buffer at a flow rate of 5 mL/min. The sample was then loaded onto the column at a flow rate of 2 mL/min, and the flow-through was collected. The column was washed with 10 column volumes of binding buffer at a flow rate of 5 mL/min. Washing with a wash buffer was performed at the same flow rate, and wash fractions were collected. Elution was done using an elution buffer at a flow rate of 2 mL/min, and eluate was collected. The fractions were analyzed by the SDS-PAGE, and the fraction with the highest purity was dialyzed in 1 × PBS, 0.1% SKL, 2 mM DTT, pH 8.8. The dialysis was conducted overnight, followed by filtration through a 0.45 μm filter, concentration, and aliquoting into 2 mL/tube, and stored at −80°C. A 12% SDS-PAGE was prepared in Tris-Gly electrophoresis buffer. The samples (10 μL) were loaded, initially run on stacking gel at 80 V for 20 min, followed by separation on resolving gel at 120 V for 60 min. Following electrophoresis, the gel was stained with Coomassie Brilliant Blue for 20 min and destained.

### Western blot analysis

After the completion of the SDS-PAGE, the gel was removed, and a nitrocellulose membrane and six pieces of 3 mm filter paper of the same size of the gel were prepared. Both the membrane and the filter papers were pre-equilibrated in chilled transfer buffer for 15 min. The transfer assembly was arranged in the following order from bottom to top: three layers of filter paper, nitrocellulose membrane, gel, and three layers of filter paper, ensuring no contact between the upper and lower filter papers. The transfer was carried out on a semi-dry electrophoretic transfer device at 15 V for 15 min to transfer the corresponding bands from the gel to the nitrocellulose membrane. After transfer, the membrane was processed for blocking, washing, incubation with primary antibody (rabbit anti-his), washing, incubation with secondary antibody (goat anti-rabbit), and washing. Finally, the nitrocellulose membrane was scanned in the Odyssey dual-color laser scanning system to obtain results.

## Results

### Confirmation of symptoms of *Nosema bombycis* infection in silkworms

**Figure 1:**
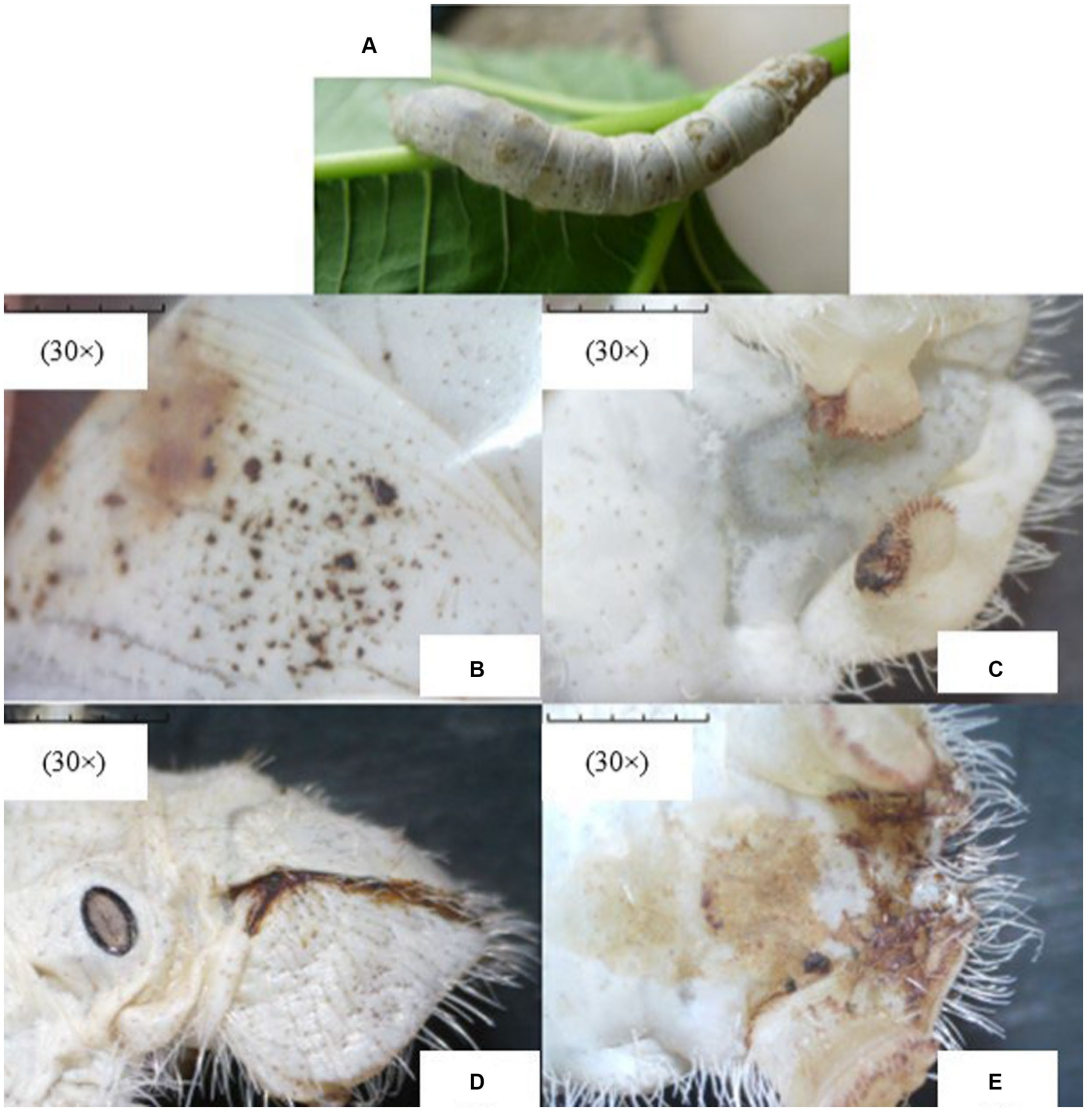
The representative images of apparent symptoms of *Nosema bombycis* infection in fifth-instar silkworms. **(A)** represents the fifth-instar silkworm infected with *Nosema bombycis*. **(B–E)** depict different symptoms of pebrine disease in the fifth-instar infected silkworms observed under the stereomicroscope. **(A)** the fifth-instar silkworm infected with *Nosema bombycis* showed many pepper-like spots on its entire body. **(B)** zoomed-in image showing pepper-like spots on the body of a fifth-instar silkworm infected with *Nosema bombycis*. **(C)** shows the tail end of a fifth-instar silkworm infected with *Nosema bombycis*, characterized by a charred black appearance and some signs of rot. **(D,E)** depict the brown secretions at the tail of a fifth-instar silkworm infected with *Nosema bombycis*.

Upon microscopic examination, the disease symptoms were quite evidently observed in infected silkworms. The representative images of the apparent symptoms observed in different parts of *Bombyx mori* infected with *Nosema bombycis* are shown in Figure 1.

### Verification and PCR amplification of *Nosema bombycis MetAP2* gene

**Figure 2:**
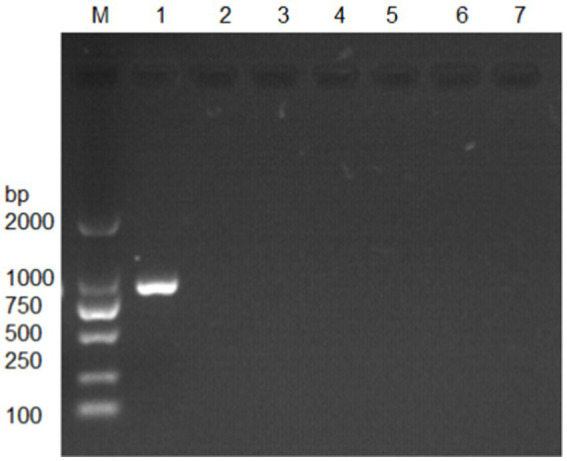
Electrophoretic gel showing amplification of *MetAP2* gene of *Nosema bombycis* (Guangdong isolate) using MC-F/R primers. Lane 1. *MetAP2* gene of *Nosema bombycis* (Guangdong isolate); Lane 2. *Bacillus bombysepticus*; Lane 3. *Bacillus thuringiensis*; Lane 4. *Beauveria bassiana*; Lane 5. BmCPV; Lane 6. BmNPV; 7. Sterilized water (negative control); M: DL2000 marker.

The *MetAP2* gene from transcriptome database of *Nosema bombycis* (Guangdong isolate was) was targeted for verification. The amplification was performed using primers MC-F/R, and the results are presented in Figure 2. The absence of band in lane 7, which served as a negative control, suggested no contamination in the reaction system. Lane 1 shows specific amplification band of *MetAP2* of *Nosema bombycis*. Lanes 2 to 6 correspond to *Bacillus bombysepticus*, *Bacillus thuringiensis*, *Beauveria bassiana*, BmCPV, and BmNPV, respectively. The PCR product of the target band (1,077 bp) was subjected to bidirectional sequencing. For determining consistency, the sequencing result was compared with the transcriptome data of *Nosema bombycis* (Guangdong isolate). The comparison showed a high consistency (99.9%). This finding confirmed that the primers MC-F/R specifically amplified *MetAP2* gene of *Nosema bombycis*.

**Figure 3:**
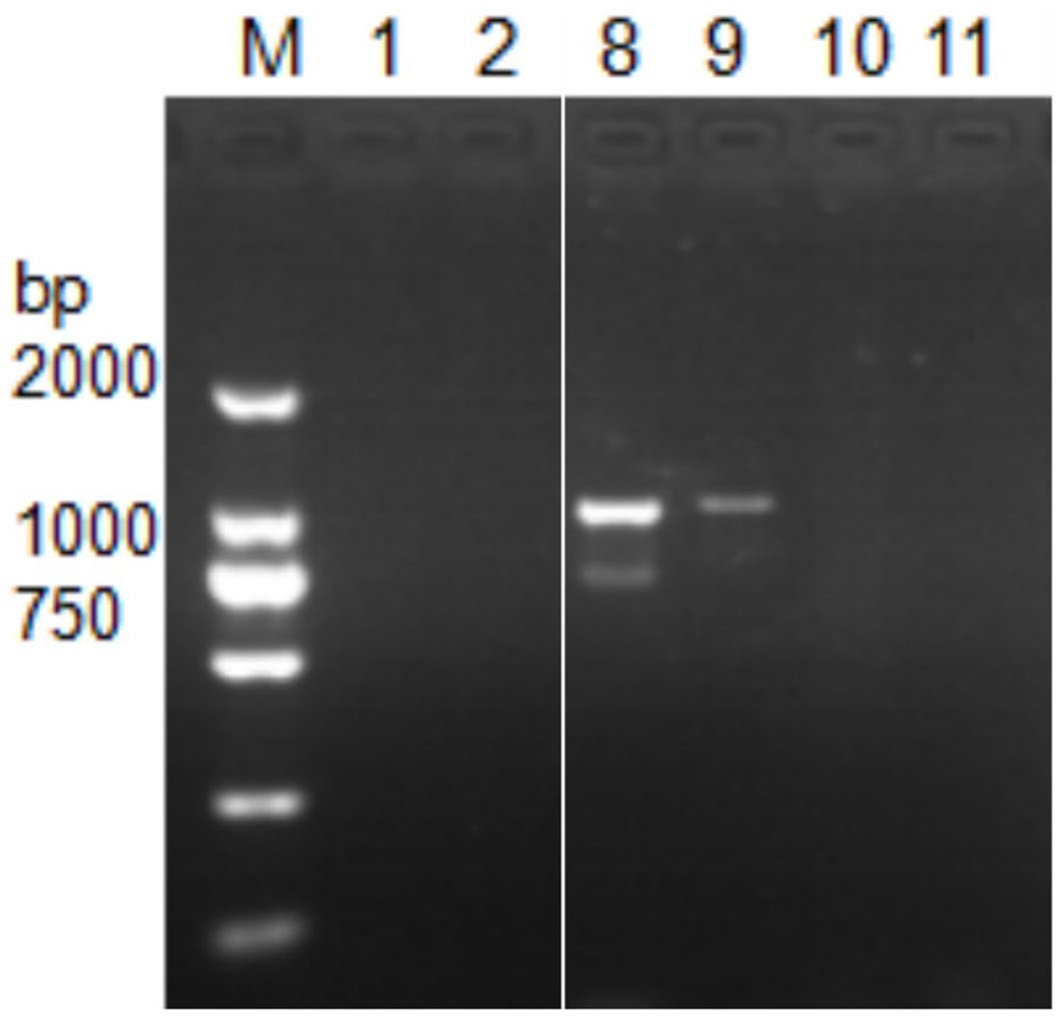
Electrophoretic gel showing PCR amplification of DNA templates of *Nosema bombycis* (Guangdong isolate) and other insect microsporidia spp., using MC-F/R primers. 1. *Endoreticulatus* sp. Zhejiang; 2. *Nosema pyraustae*; 8. *Nosema antheraeae*; 9. *Nosema bombycis*; 10. Midgut of silkworm; 11. Sterilized water; M: DL2000 Marker. Lanes 3 to 7 in this gel represented *MetAP2* genes of other microsporidia spp. of mulberry pests. These lanes were removed, as they were not part of the current manuscript. White border separates the two cropped segments of gel.

Next, DNA of *Nosema bombycis*, *Nosema antheraeae*, *Endoreticulatus* sp. Zhejiang, and *Nosema pyraustae* were used for amplification of *MetAP2* gene. DNA of healthy silkworm midgut was used as a template. The electrophoresis results are shown in Figure 3. No bands were observed in Lanes 1 (*Endoreticulatus* sp. Zhejiang) and 2 (*Nosema pyraustae*), indicating that *MetAP2* gene of these microsporidia spp. was not amplified with the primers used in the present study. Lanes 10 and 11, DNA of the midgut of a healthy silkworm and sterile water did not show amplification bands, indicating that the system was free of contamination. Lanes 8 and 9, representing *MetAP2* genes *of Nosema bombycis* and *Nosema antheraeae*, respectively yielded corresponding bands (Figure 3). The PCR products were recovered and bidirectional sequencing was performed. The sequencing results were compared for consistency with the transcriptome data of *Nosema bombycis* and *Nosema antheraeae* (Qazi and Liu, 2024; unpublished results). The comparison results of GC content are shown in Table 1.

**Table 1:** Sequencing length (bp) and GC content (%) of *MetAP2* genes of *Nosema bombycis* (Guangdong isolate) and *Nosema antheraeae* amplified in the present study.

*Nosema* species	Length (bp)	GC content (%)
*Nosema bombycis*	1,077	33.24
*Nosema antheraeae*	1,068	32.21

DNA templates of *Endoreticulatus* sp. Zhejiang and *Nosema pyraustae* were not amplified.

### Homology and phylogenetic analyses of MetAP2 of *Nosema bombycis*

#### Analysis of *MetAP2* gene sequences of *Nosema bombycis* and other insect microsporidia spp.

**Figure 4:**
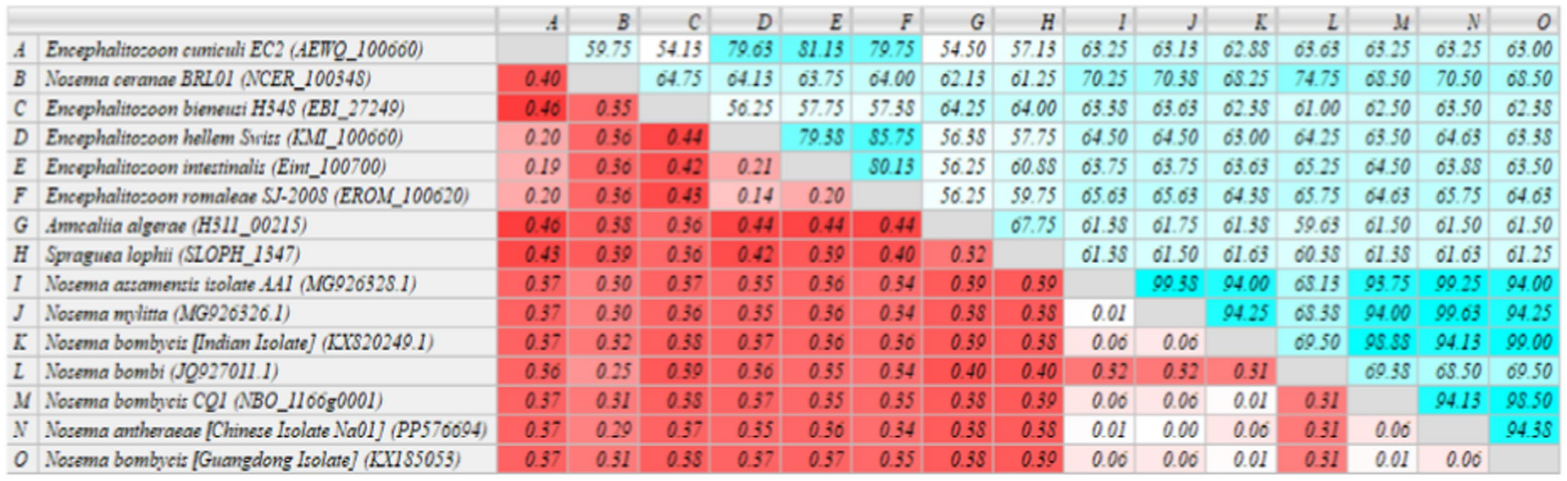
Homology comparison of *MetAP2* genes between *Nosema bombycis* (Guangdong isolate) and other microsporidia spp. Values in turquoise colored boxes denote the percent (%) identity, and values in red colored boxes denote the divergence. Color gradient highlights the values (%identity and divergence) from higher (dark) to lower (light) range. *MetAP2* genes of *Nosema bombycis* (Guangdong isolate) and *Nosema antheraeae* (Chinese isolate) were sequenced in the present study.

The sequenced *MetAP2* gene *of Nosema bombycis*, along with *MetAP2* genes from other select microsporidia spp. were analyzed for homology and used to construct RAxML phylogenetic tree. The homology comparisons (Figure 4) revealed that the homology (%identity) between *MetAP2* genes of different microsporidia spp. ranged from 54.13 to 99%. Specifically, the homology between *Nosema bombycis* (Guangdong isolate) and other microsporidia spp. ranged from 61.25 to 99%. *Nosema bombycis* (Guangdong isolate) sequenced in the present study had the closest %identity with *Nosema bombycis* (Indian isolate; 99%) and *Nosema bombycis* (CQ1; 98.50%). Meanwhile, %identity with Nosema spp. infecting wild silkworm spp. ranged between 94 to 94.38%. From these, the closest relative to *Nosema bombycis* (Guangdong isolate) was found to be the Chinese oak silkworm microsporidium (*Nosema antheraeae*; sequenced in the present study), with a %identity of 94.38% (Figure 4).

**Figure 5:**
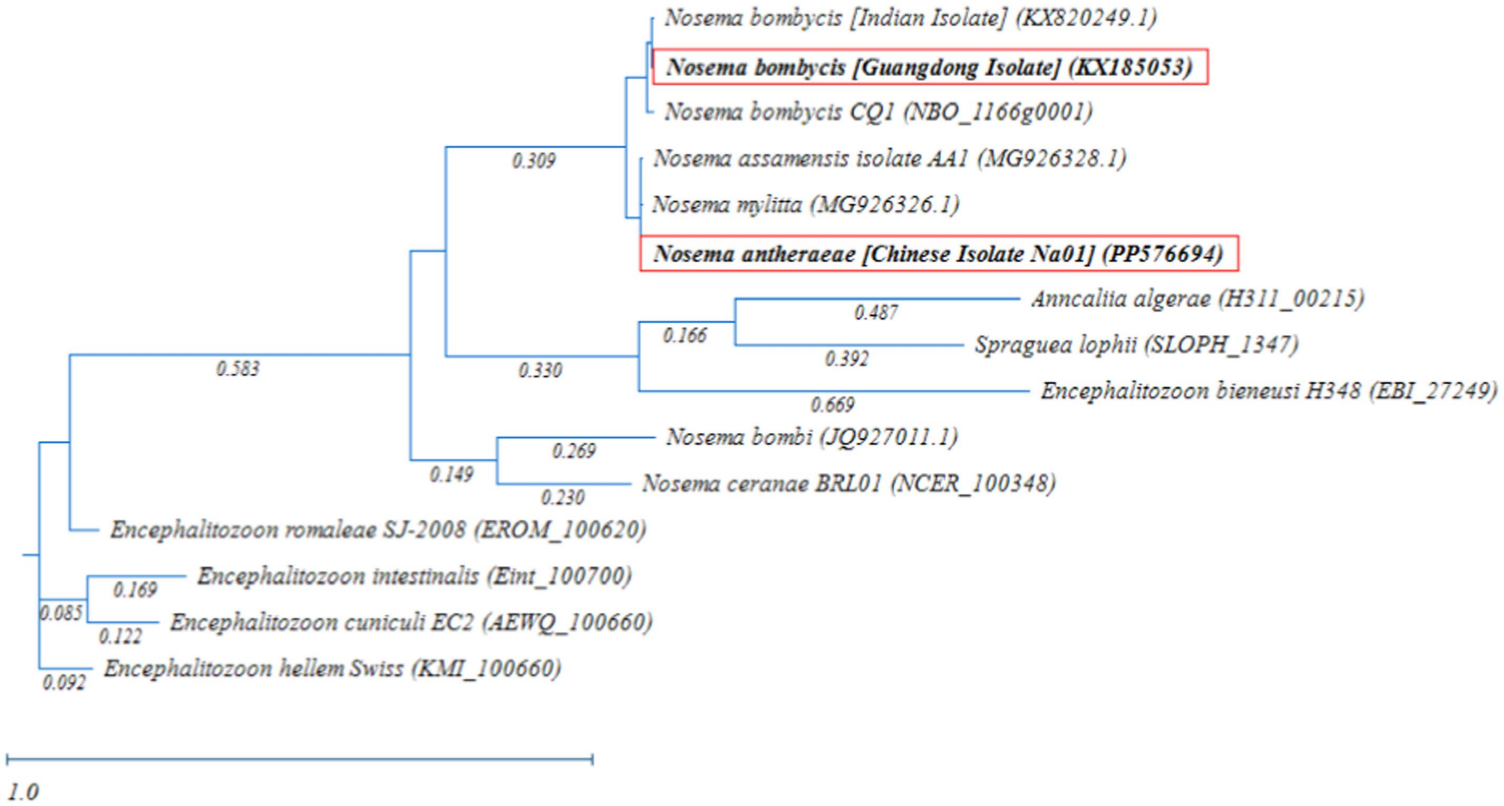
Randomized Axelerated Maximum Likelihood (RAxML) phylogenetic tree of *MetAP2* gene sequences of *Nosema bombycis* (Guangdong isolate) and other microsporidia spp. Red boxes highlight *MetAP2* genes of *Nosema bombycis* (Guangdong isolate) and *Nosema antheraeae* (Chinese isolate) that were sequenced in the present study. The scale bar indicates sequence divergence.

Meanwhile, the phylogenetic analysis based on RAxML tree showed that *Nosema bombycis* sp. including the Guangdong isolate clustered together, with Nosema sp. of the domestic and wild silkworms forming separate branches (Figure 5). The microsporidia spp. *Nosema ceranae* (honeybees) *and Nosema bombi* (bumblebees) clustered on the same branch and had a distant relationship with the *Nosema bombycis* (Guangdong isolate) and *Nosema antheraeae* sequenced in the present study.

In addition, microsporidia of *Nosema* spp. showed a more distant relationship with members of Encephalitozoon genus, in which many spp. infecting humans formed a separate major branch (Figure 5). Within the Encephalitozoon genus, *Encephalitozoon cuniculi*, *Encephalitozoon intestinalis*, *Encephalitozoon hellem*, and *Encephalitozoon romaleae* showed a close relationship between them (Figure 5). *Anncaliia algerae* (human isolate) and *Spraguea lophii* (Monkfish microsporidium), formed a separate branch and showed more distant relationship with other species (Figure 5). The results of the homology comparisons were consistent with the outcomes of the phylogenetic analysis.

#### Homology and phylogenetic analyses of MetAP2 amino acids sequences of different species

The homology comparisons revealed that the homology (%identity) between MetAP2 of *Nosema bombycis* (Guangdong isolate) and other spp. ranged from 40.18 to 96.93% (Supplementary Figure S2).

**Figure 6:**
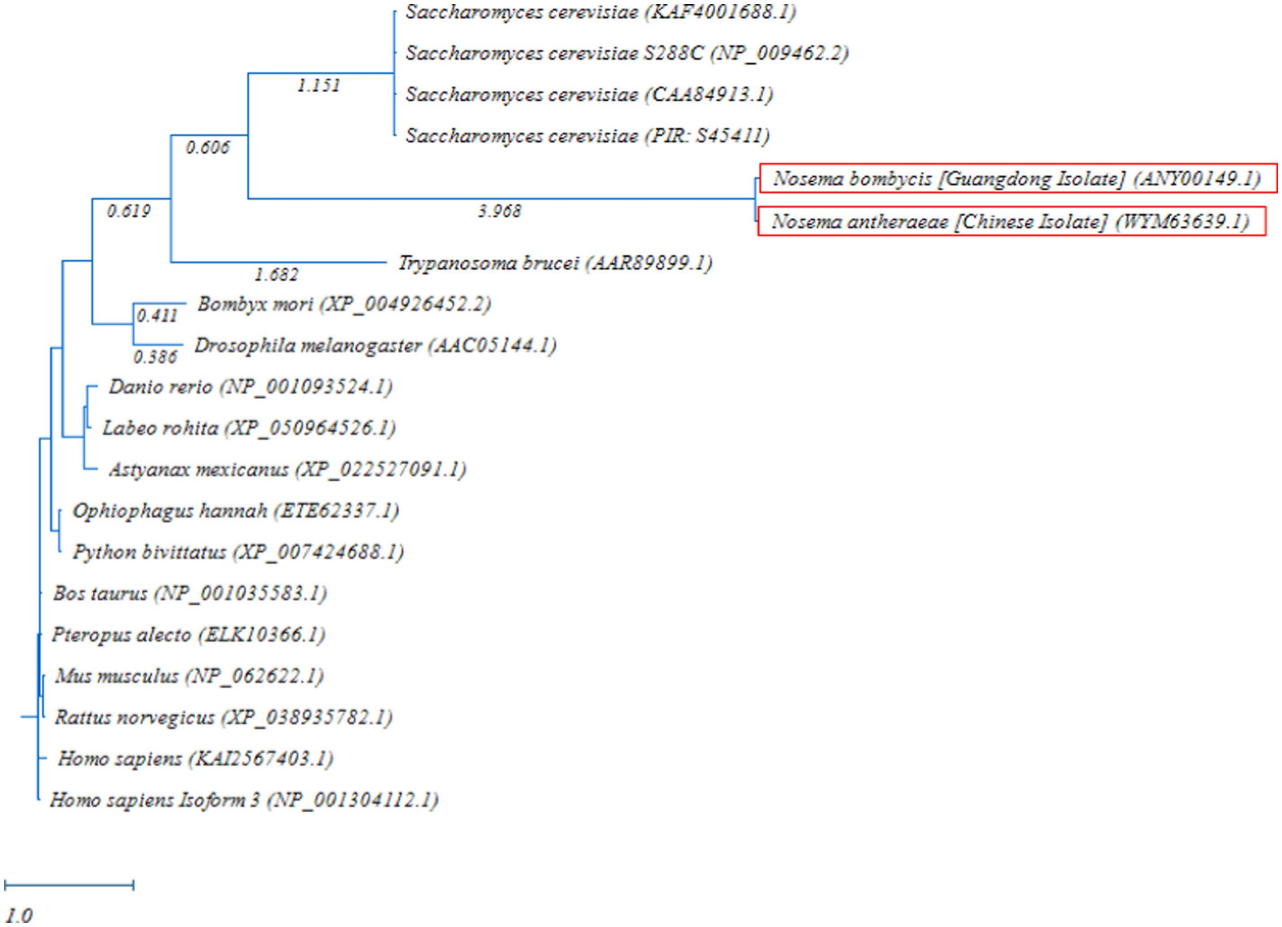
Randomized Axelerated Maximum Likelihood (RAxML) phylogenetic tree of *MetAP2* amino acids sequences of *Nosema bombycis* (Guangdong isolate) and other species. Red boxes highlight amino acid sequences of *MetAP2* of *Nosema bombycis* (Guangdong isolate) and *Nosema antheraeae* (Chinese isolate) that were generated in the present study. The scale bar indicates sequence divergence (i.e., amino acid substitution per site).

The phylogenetic comparison of MetAP2 protein of different species was carried out by constructing a RAxML tree. As shown in Figure 6, the MetAP2 amino acids of chordates clustered in one group, with mammals showing close evolutionary proximity. The MetAP2 amino acids of insects and microorganisms were found to be relatively close. Specifically, insects (*Bombyx mori* and *Drosophila melanogaster*) clustered on the same branch and formed a distinct group, while *Saccharomyces cerevisiae*, *Nosema bombycis*, *Nosema antheraeae*, and *Trypanosoma brucei* did not form a single group, but they were relatively close in the distance.

#### Homology and phylogenetic analyses of MetAP2 amino acid sequences between *Nosema bombycis* (Guangdong isolate) and its relatives Aspergillus spp., and *Saccharomyces cerevisiae*

**Figure 7:**
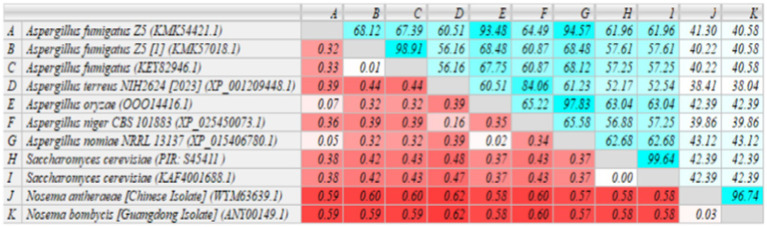
MetAP2 amino acid sequence alignment results of *Nosema bombycis* (Guangdong isolate), *Aspergillus* spp., and *Saccharomyces cerevisiae*. Values in turquoise colored boxes denote the percent (%) identity, and values in red colored boxes denote the divergence. Color gradient highlights the values (%identity and divergence) from higher (dark) to lower (light) range.

The homology of MetAP2 amino acid sequences between *Nosema bombycis*, *Nosema antheraeae*, *Aspergillus* spp., and *Saccharomyces cerevisiae* was analyzed. As shown in Figure 7, the percent identity of MetAP2 amino acids ranged between 38.04 and 96.74%, with the percent identity between *Nosema bombycis* and different *Aspergillus* spp. not being particularly high (38.04 to 43.12%).

**Figure 8:**
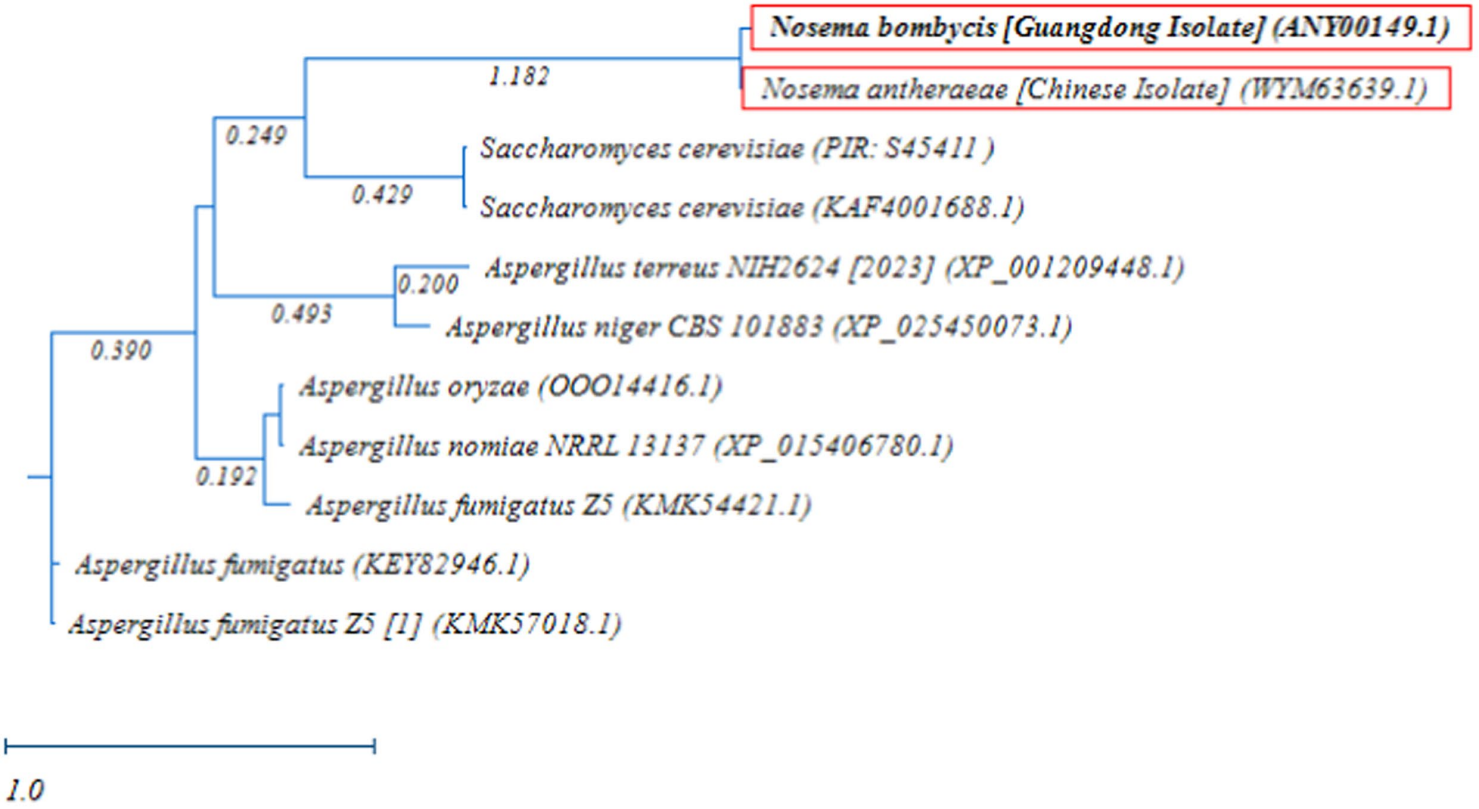
Randomized Axelerated Maximum Likelihood (RAxML) phylogenetic tree of MetAP2 amino acids sequences of *Nosema bombycis* (Guangdong isolate), *Nosema antheraeae, Aspergillus* spp., and *Saccharomyces cerevisiae*. Red boxes highlight amino acid sequences of MetAP2 of *Nosema bombycis* (Guangdong isolate) and *Nosema antheraeae* (Chinese isolate) that were generated in the present study. The scale bar indicates sequence divergence (i.e., amino acid substitution per site).

Next, the RAxML phylogenetic tree was constructed using the MetAP2 amino acid sequences of *Nosema bombycis* (Guangdong isolate), *Nosema antheraeae*, *Aspergillus* spp., and *Saccharomyces cerevisiae*. As shown in Figure 8, as expected, MetAP2 of *Nosema bombycis* and *Nosema antheraeae* clustered together and formed a distinct branch, which was separated from the branches of *Aspergillus* and *Saccharomyces cerevisiae* spp. In summary, the distant relationship as seen in these molecular evolutionary (phylogenetic) analyses altogether signifies that the conservation and evolutionary relationships of MetAP2 gene and amino acids sequences are closely linked to the species relationships.

### Bioinformatics analysis of *Nosema bombycis* MetAP2 gene and protein

The full length of *Nosema bombycis MetAP2* DNA sequence was found to be 1278 base pairs (bp). This included a 5′ non-coding region of 49 bp, a 3′ non-coding region of 152 bp, and an open reading frame of 1,077 bp, encoding a total of 358 amino acids. The predicted molecular weight of MetAP2 protein was found to be 40.51 kDa approximately, with a molecular formula of C_1816_H_2845_N_479_O_542_S_14_. The theoretical isoelectric point was 5.91, with an Aliphatic index of 88.24, instability index of 31.64, and the Grand average hydropathicity (GRAVY) index of −0.337, all indicating characteristics of a stable protein.

The conserved structural domain of MetAP2 protein is depicted in Supplementary Figure S3. Briefly, the predicted conserved region of *Nosema bombycis* MetAP2 protein spanned approximately 60–355 amino acids, with predicted active sites located at positions 110, 130, 140, 210, 241, and 340.

The results of predictions of transmembrane domains and signal peptides in *Nosema bombycis* (Guangdong isolate) MetAP2 protein are shown in Supplementary Figures S4, S5, respectively. Briefly, these analyses showed absence of transmembrane domains and signal peptides in predicted *Nosema bombycis* MetAP2 protein.

#### Prediction of secondary structural domains of MetAP2 protein

The prediction of secondary structural domain of MetAP2 protein is shown in Supplementary Figure S6. The predicted protein had a predominance of alpha-helix (Alphahelix) structural elements, consisting of 115 amino acids. Additionally, random coil structural elements consisting of 181 amino acids were also present. Given the presence of typical alpha-helix structural elements in MetAP2 protein structure, it can be hypothesized that the *Nosema bombycis* MetAP2 protein may form certain structural or functional domains.

#### Prediction of subcellular localization of MetAP2 protein

Based on online prediction software PSORT II, the prediction of subcellular localization of MetAP2 protein revealed the following descriptions: 1. the sequence has a potential cleavage site between positions 54 and 55. R-2 motif at 12 MRP|IV was found at mitochondrial presequence cleavage sites. No prenylation modification motifs were present. 2. The sequence lacks an N-terminal signal peptide and N-myristoylation patterns. There were no C-terminal retention motifs and C-terminal glycosylation signals like SKL, SKL2, or peroxisomal targeting signals. 3. No endoplasmic reticulum (ER) retention motif was present in the C-terminus. There were two motifs (XXRR-like motif in the N-terminus: RPIV and KKXX-like motif in the C-terminus: KGDD) in the ER membrane retention signals were predicted. No predicted vacuolar targeting motifs were present in the sequence. Additionally, there were no motifs for transport from the cell surface to the Golgi apparatus, and the tail end of the sequence lacked tyrosine and Dileucine motifs. 4. The sequence did not contain RNA-binding motifs, and either type of Actinin-type actin-binding motifs. No DNA binding and ribosomal protein motifs were present. 5. Lupas’s algorithm detected no coiled-coil regions, suggesting a cytoplasmic/nuclear localization. NUCDISC, which identifies nuclear localization signals, found no binding or free pat4 and pat7 motifs, with virtually zero residual content and an NLS index of −0.47. Reinhardt’s method predicted that the MetAP2 was located in the cytoplasm, with a reliability sore of 76.7. The sub-cellular localization results retrieved through DeepLoc - 2.0 were consistent with PSORT II and showed a probability value of 0.7329 for cytoplasmic localization.

### Homology modeling-based 3D structure of MetAP2 protein of *Nosema bombycis*

**Figure 9:**
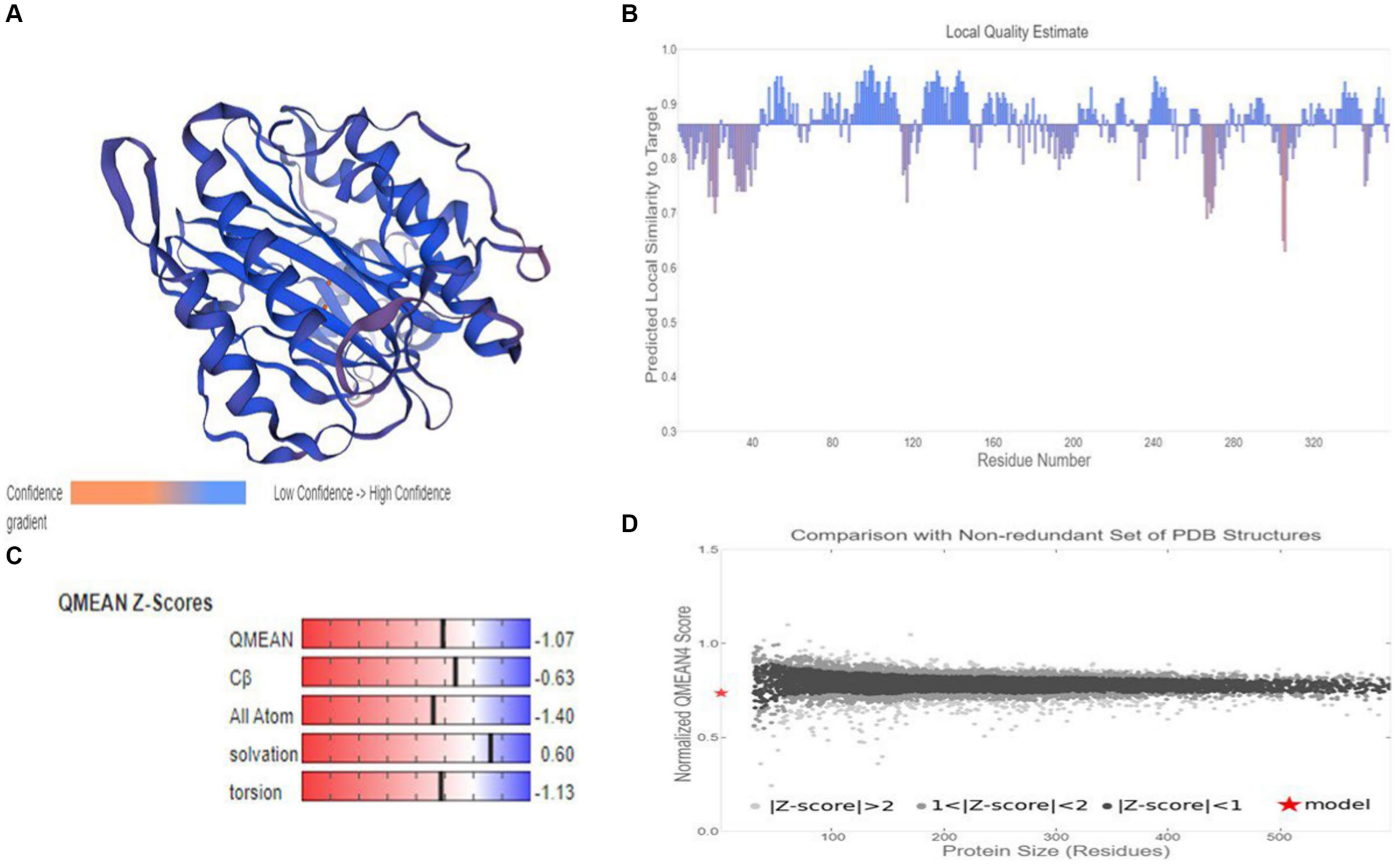
Homology modelling of MetAP2 protein of *Nosema bombycis* (Guangdong isolate). **(A)** 3D structure of MetAP2 protein of *Nosema bombycis*. This model was developed based on template 3 fm3.1 (MetAP2 *Encephalitozoon cuniculi*). Color scheme of ribbon represents the confidence gradient (key shown). Two orange spheres represent ligand [metal ions; FE (III)] coordinated within the active site. **(B)** QMEAND is local score: this shows, for each residue of the model (reported on the x-axis), the expected similarity to the native structure (y-axis). Typically, residues showing a score below 0.6 are expected to be of low quality. Different model chains are shown in different colors. **(C)** QMEAN Z-score: Z-scores around 0.0 therefore reflect a “native-like” structure and, as a rule of thumb, a “QMEAN” Z-score below −4.0 indicates a model with low quality. **(D)** Comparison plot: the x-axis shows protein length (number of residues). The y-axis is the “QMEAN” score. Every dot represents one experimental protein structure. Black dots are experimental structures with a “QMEAN” score within 1 standard deviation of the mean (|Z-score| between 0 and 1), experimental structures with a |Z-score| between 1 and 2 are grey. Experimental structure that are even further from the mean are light grey. The actual model is represented as a red star.

Based on target-template alignment, two homology models (models 1 and 2) were built. The 3D structures of models 1 and 2 are presented in confidence scheme view (Figure 9A; Supplementary Figure S7A). The overall model quality measurement evaluation showed that the Global Model Quality Estimate (QMQE) value for models 1 and 2 was 0.97 and 0.89 respectively, indicating the high accuracy and reliability of the developed models. The QMEANDisCo global score of model 2 was 0.86 ± 0.05. The QMEANDisCo local score, QMEAN Z-score and comparison plot for model 2 are shown in Figures 9B–D. These analyses were not computed for model 1, as its template (A0A3G6ILP9.1.A) itself was built using AlphaFold2 database. The Ramachandran Favoured value for models 1 and 2 were 95.48 (Supplementary Figure S7B) and 97.47% (Supplementary Figure S7C), respectively. The target-template sequence alignments for models 1 and 2 are presented in Supplementary Figures S7D,E. Overall, model 2 (Figure 9A) was considered as practically more reliable structure, as it was based on template sequence of experimentally produced MetAP2 of *Encephalitozoon cuniculi* (ID: 3 fm3.1.A).

### Prokaryotic expression of MetAP2 protein

#### Construction of cloning vector

After the PCR amplification of *Nosema bombycis* DNA using primers MC-F/R, the product was subjected to agarose gel electrophoresis and subsequent gel recovery (Supplementary Figure S8). The lane 3, representing sterile water, displayed no band amplification, indicating no contamination in the reaction system. The MC-F/R primers also did not produce any nonspecific dimer amplification. Bands in lanes 1 and 2 were approximately 1,000 bp, clear and bright, making them suitable for further cloning and expression experiments.

Following PCR amplification and purification of *MetAP2* gene, the cloning was performed. The PCR identification of bacterial liquid cultures from individual colonies is depicted in Supplementary Figure S9. The lane 4, representing sterile water, showed no amplification band, confirming no contamination in the reaction system. Although Lane 3 showed nonspecific amplification in the midgut of silkworm but did not affect the results of this experiment. Lane 1, representing *Nosema bombycis* DNA, showed a specific amplification band at around 1,100 bp, indicating a well-functioning reaction system. Lane 2 showed a specific amplification band at the corresponding position for the post-cloning bacterial liquid, suggesting the successful construction of *MetAP2* gene cloning vector.

Then, the recovered target fragment was ligated with pMD19-T vector and transformed into DH5α cells. Positive clones were sent to Sangon Biotech (Shanghai) Co., Ltd. for sequencing verification. The correct pMD19-T recombinant plasmids were selected after nucleotide sequence comparison.

### Construction of pET-28A–met

The correctly sequenced pMD19-T recombinant plasmid was subjected to double digestion, and the digested fragment was ligated with the prokaryotic expression vector pET-28A(+). This construct was then transformed into *Rosetta* (DE3) cells to obtain positive recombinant plasmids. The PCR and double digestion with NdeI and XhoI for verification are presented in Supplementary Figure S10. The results indicate that the size of the enzyme-digested bands corresponds to *MetAP2* gene, demonstrating the successful construction of prokaryotic recombinant plasmid pET-28A–met. This allowed for the subsequent expression of protein.

### Induction of protein expression

Next, the SDS-PAGE analysis was conducted which showed the presence of a specific target band at ~43–45 kDa (Supplementary Figure S11). Although, this observed size was slightly higher than the predicted size, it was sufficient to indicate the successful expression.

### Nickel nitrilotriacetic acid (Ni-NTA) agarose affinity chromatography purification of protein

The SDS-PAGE results after purification using Ni-NTA agarose affinity chromatography are depicted in Supplementary Figure S12. After ultrasonic disruption, the expressed protein was present both in the supernatant and the pellet, with a higher concentration in the supernatant. This allowed for the use of the supernatant from the induced expression of the recombinant bacteria for affinity purification. A volume of 1 mL of the protein supernatant was subjected to purification through a nickel affinity column. The subsequent 12% SDS-PAGE electrophoresis demonstrated a high purity of the purified protein (Supplementary Figure S12: lanes 4 and 5). The results indicated a significant increase in the expression of the soluble protein. The purified product, analyzed through SDS-PAGE, showed a single protein band around ~43–45 kDa, indicating the effectiveness of the purification method in yielding a high-purity protein (Supplementary Figure S12: lanes 4 and 5).

### Identification, purification and expression of protein

**Figure 10:**
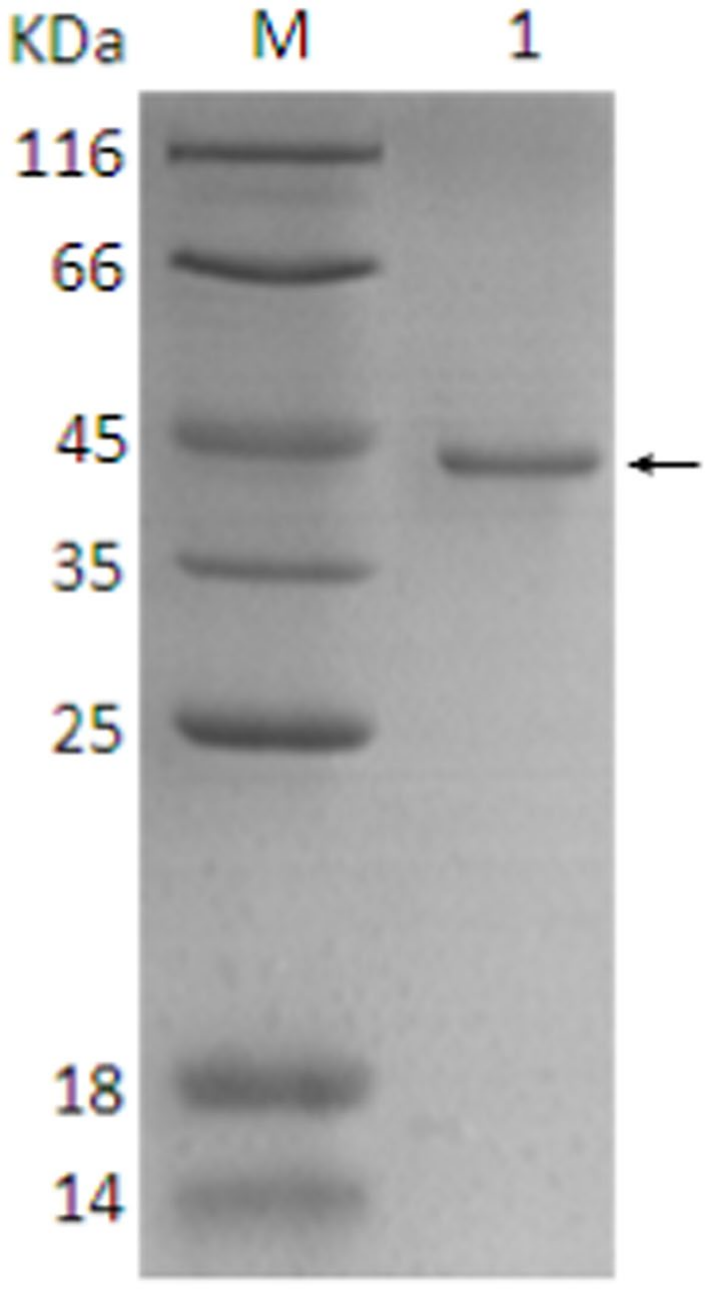
SDS-PAGE analysis of final protein purification. 1. Target protein (pointing arrow); M: Protein marker.

**Figure 11:**
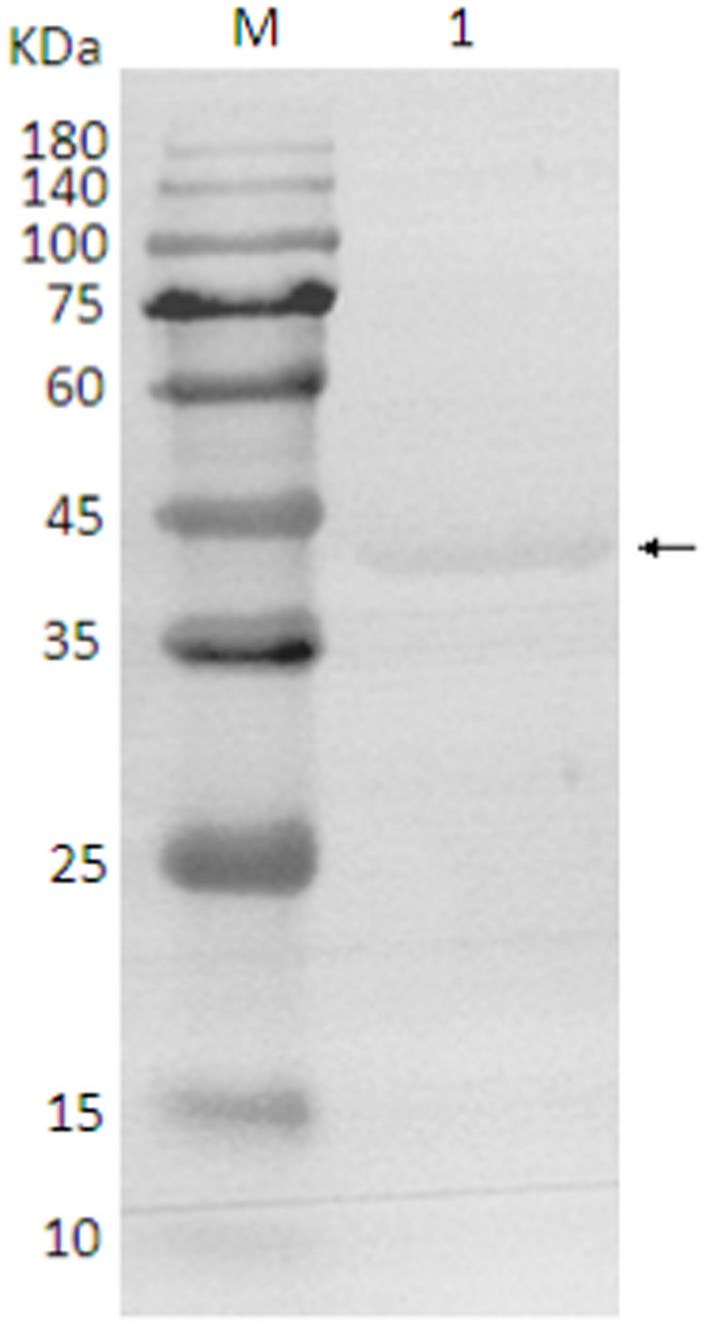
Western blot analysis of protein. 1. target protein (pointing arrow); M: pre-stained marker.

The SDS-PAGE of the protein is shown in Figure 10, and the Western blot analysis result are presented in Figure 11. The purified protein showed a clear band at the corresponding position on SDS-PAGE (Figure 10), indicating the successful purification. As shown in Figure 11, the Western Blot analysis showed a target band at around ~43–45 kDa. The Western blot identification indicated that the protein can specifically bind with the anti-His tag antibody, demonstrating good immunoreactivity, and confirming that the protein was successfully expressed in *E. coli* Rosetta (DE3).

## Discussion

It has been argued that, a thorough understanding of essential genes related to the survival of a given pathogen is critical in devising a viable control strategy (26). This becomes increasingly important while determining therapeutic options and diagnostic tools for fascinating pathogens like microsporidia. Pebrine, the only disease with a mandatory quarantine (22), is a devastating disease of domesticated silkworms with a huge economic impact on sericulture industry in countries like China. This disease is caused by *Nosema bombycis*, for which currently there are no established therapeutic and control options available. Although, our understanding of the mechanisms of high infectious ability and the broad host range of *Nosema bombycis* is limited so far, it has been previously argued that certain factors such as gene duplication, transposable element expansion, and horizontal gene transfer are the likely contributors to high pathogenicity of this mysterious parasite (48). Based on genomic evidence, it was suggested that the specific essential genes of *Nosema bombycis* maybe utilized as primary targets while devising therapeutic (48) and diagnostic (26) options for this deadly disease.

Historically, MetAP2 is known as a bifunctional protein (49). It has been known for its peptidase activity and involvement in inhibition of phosphorylation of eukaryotic initiation factor 2α in yeast and mammals (49, 50). But given the fact that polylysine stretch was found to be missing from the NH2-terminus in MetAP2 of microsporidia spp., it lacked the ability to inhibit phosphorylation of initiation factor2α (50). However, it was argued that this lack of N-terminal extension (polylysine rich region) was not associated with enzyme activity of MetAP2 (51). It was reported that MetAP2-dependent yeast *Saccharomyces cervisiae* was killed by ovalicin, an analogue of fumagillin, whereas MetAP1-dependent yeasts were not killed (49, 52). This evidence indicated that MetAP2 is a selective cellular target of fumagillin and its analogues (23, 51). We know that both *MetAp1* and *MetAP2* are present in higher eukaryotes and yeasts (51). But as with the other microsporidian spp. (50), our transcriptome data (Qazi and Liu, 2024; unpublished results) returned only *MetAP2* both in *Nosema bombycis* (Guangdong isolate) and *Nosema antheraeae*. Given that *MetAP2* gene is currently the only known cellular target of fumagillin and its analogues (23, 50, 51), in the present study we performed an in-depth molecular and phylogenetic characterization of MetAP2 gene and protein of the Guangdong isolate of *Nosema bombycis*. Meanwhile, MetAP2 of *Nosema antheraeae* was also sequenced and annotated for phylogenetic comparison.

To our knowledge, this is the first report of identification, expression, characterization, and phylogenetic analysis of MetAP2 gene and its protein in the Chinese (Guangdong) isolate of *Nosema bombycis*. Utilizing the transcriptome data (Qazi and Liu, 2024; unpublished results) of Guangdong isolate of *Nosema bombycis* and the sequence resources generated in this study, it was observed that the full length of the DNA sequence of *MetAP2* gene was 1,278 bp, including a 5′ non-coding region of 49 bp, a 3′ non-coding region of 152 bp. Specifically, a large open reading frame of 1,077 bp, encoding 358 amino acids, was found. The predicted molecular weight of MetAP2 protein was 40.5 kDa, but the observed molecular weight obtained through prokaryotic expression and purification was about ~43–45 kDa, which was slightly higher than the predicted value in the present study. This phenomenon is not uncommon and has been reported for several proteins studied previously [Guan et al. (53) and references therein]. The GC content (35.48%) of *MetAP2* gene of *Nosema bombycis* was comparable with the lower GC% as observed for its wild relative *Nosema antheraeae* studied in the present study. Tellingly, the overall GC content of *Nosema bombycis* (Guangdong isolate) and *Nosema antheraeae* (Chinese isolate) transcriptome was also observed to be 31 and 28%, respectively (Qazi and Liu, 2024; unpublished results). This is particularly relevant because the lower GC content has been linked to a larger proportion of repetitive sequences relative to the total assembled sequence and longer intergenic regions (54). Additionally, there is an evidence by Pan et al., in their landmark paper (48), where they have shown that *Nosema bombycis* genome is composed of over 38% repetitive elements.

Although, limited work has been done on *MetAP2* gene of *Nosema bombycis*, there is a recent report (55) on characterization of *MetAP2* gene of the Indian isolate of *Nosema bombycis*. To compare the sequence identity between the Guangdong and the Indian isolates of *Nosema bombycis*, we performed the BLAST search analysis using the NCBI (see text footnote 3), and observed a high sequence identity (99%) between the two isolates, with a difference only at 10 bp (1,067/1077) in the open reading frame. Similarly, we also compared sequence identity between *Nosema bombycis* (Guangdong isolate) and uncharacterized *MetAP2* sequence of *Nosema bombycis CQ 1* (Chongqing, China) isolate that was retrieved from its genome sequence data set (48). Interestingly, BLAST search analysis revealed a difference of 27 bp (1,050/1077) in the open reading frames of the two sequences. In keeping with these differences, we currently do not know whether these subtle variations in *MetAP2* gene sequences between *Nosema bombycis* isolated from geographically different locations have any biological significance. But, in keeping with the local context, due consideration is still required while designing of therapeutic or diagnostic options. In any case, further in-depth molecular studies are still required to gain more insight in this regard.

Previously, it was shown that indeed intraspecies polymorphism does exist between four different isolates of *Nosema bombycis* recovered from different geographical regions of China, including one from Guangdong province (56). These authors further reported that the obvious polymorphic differences between analogous sequences were seen both in terms of length and composition of sequence. In a study on *Nosema* spp. of honeybees in Saudi Arabia (57), it was reported that 16S rRNA gene sequence analysis of two geographically different isolates (*ksuNC4* and *ksuNC6*) showed a high identity 99% (217/218) between sequences. In this case, the difference was only found at one position, i.e., the 18th bp was polymorphic with one gap. In the same study, a 100% sequence identity of the Saudi isolate (*ksuNC4*) of *Nosema ceranae* with the 16S rDNA of other previously reported isolates from different parts of the world was reported (57). It was argued that this identity in sequences can be attributed to the evolutionary relationship between different isolates and species (57). Previously, two putative isolates of *Nosema* sp. (PX1 and PX2) that were recovered from the diamondback moths in Taiwan also showed high divergence in the sequences of the ITS and IGS regions (58). Recently, based on the IGS sequence analysis, 12% average sequence variation was reported in 20 isolates of *Nosema mylitta* collected from different geographic locations in India (59). Based on the genetic and phylogenetic analysis, it was reported that the evolutionary divergence in *Nosema mylitta* was potentially associated with adaptation of pathogen, host defense response, and the geographic conditions in which the host lives (59). This molecular evidence augments the argument that the variation in gene sequences in microsporidian spp. is indeed a result of evolutionary process (59).

Due to the complex silkworm rearing environment and management practices of mulberry orchards, it is reasonable to argue that the cross-infection [e.g., as reported in Huang et al. (60)] of pathogenic insect microsporidian spp. is difficult to control in sericulture production settings. In keeping with the context of the present study, it is pertinent to mention here that the biological evolutionary relationship between *MetAP2* of *Nosema bombycis* of silkworms and other microsporidian spp. carried by different mulberry orchard pests has not been reported yet. It is therefore desirable that the future studies should cover this caveat.

In the present study, homology comparisons showed a varied percent identity between *MetAP2* gene of *Nosema bombycis* and that of other insect and human microsporidian spp. Tellingly, *Nosema bombycis MetAP2* showed a close relationship (%identity) with *MetAP2* of *Nosema antheraeae* of *the* Chinese oak silkworms, and *Nosema mylitta* and *Nosema assamensis*, both infecting the Indian wild silkworms (59, 61). Consistent with the homology comparison, the phylogenetic analysis showed that *MetAP2* of *Nosema bombycis* clustered together and formed a separate branch, with a close evolutionary relationship with *Nosema* spp. of wild silkworms *Nosema antheraeae*, *Nosema mylitta* and *Nosema assamensis*. *Nosema ceranae* and *Nosema bombi* clustered together and showed a distant relationship with *MetAP2* gene of *Nosema bombycis* in the phylogenetic tree. Understandably, *MetAP2* of human microsporidia spp. clustered on the same branch, showing a close relationship between them, but showed a distant evolutionary relationship with *Nosema* spp. of domestic and wild silkworms. Previously, it was argued that *Nosema* spp. infecting domestic and wild silkworms are regarded as “True *Nosema* group/clade,” whereas those infecting Lepidoptera and other insects such as honeybees and bumblebees are regarded as “*Nosema*/Vairimorpha group/clade” (9, 61). In the present study, *MetAP2* of *Nosema* spp. of domestic and wild silkworms showed a close phylogenetic relationship between them, but were placed distantly from *Nosema ceranae* (honeybees) and *Nosema bombi* (bumblebees).

The phylogenetic analysis of MetAP2 proteins of various species revealed that amino acids sequences of MetAP2 of *Nosema* spp. did not cluster with any other organisms, but they were placed closer in distance to *Saccharomyces cerevisiae* and *Trypanosoma brucei*. Similarly, further homology and phylogenetic analyses of MetAP2 amino acid sequences between *Nosema* spp., and its ancestral fungal relatives *Aspergillus* spp., and *Saccharomyces cerevisiae* returned consistent results.

Due to lack of available sequences, there has been a limited use of protein coding genes in phylogenetic reconstruction of microsporidian spp. (62). It has been said that *MetAP2* genes of microsporidian spp. do not show any stronger relationship to fungal *MetAP2* than that of other eukaryotes (51). Based on phylogeny of *MetAP2*, it was previously shown that human microsporidia lineage did not cluster with fungi *Aspergillus* and *Saccharomyces* spp. (62). These findings indicate that the conservation and evolutionary relationships of MetAP2 amino acids are closely linked to the species relationships. The molecular weight of MetAP2 protein of *Nosema bombycis* observed in the present study was also closer (~43–45 kDa) to the molecular weights of *Encephalitozoon* spp. of microsporidians (48 to 49 kDa) and *Bruchiolu algerae* (47 kDa) reported previously (50, 63). To put this in the context, the molecular masses of MetAP2 proteins of fungi *Saccharomyces cerevisiae* and humans were reported to be much higher, i.e., 65 and 67 kDa, respectively (50).

It has been said that “*any level of physical characterization of a protein, as opposed to its absence, is valuable*” (64). In recent times, 3D structure modelling of proteins is seen as a focal point where diverse research efforts can combine to provide a detailed view of the underlying mechanistic basis (65). Homology modeling is now seen as important utility in biomedical research, particularly making the targeted drug discovery research faster, easier, cheaper and more practical (66, 67). The selection of an appropriate structural template is a prerequisite in successful application of 3D model of protein. However, templates with the highest sequence identity to the target protein does not necessarily mean that they are appropriate choices (65). It is usually believed that close homologue structures can produce accurate models; however, templates with lower sequence similarity (~20%) can still produce suitable models (68, 69). In keeping with this notion, we developed two 3D structures models of MetAp2 protein. Model 1 was based on template of MetAp2 of *Nosema assamensis* (ID: A0A3G6ILP9.1.A) and had high quality estimate values. But, the template itself was prepared using a prediction (AlphaFold2) database. We therefore prepared a second model based on template of MetAp2 of *Encephalitozoon cuniculi* (ID: 3 fm3.1.A). Although, template used in model 2 had a lower sequence identity with the target sequence, it was considered more reliable, as it was experimentally produced using x-ray crystallography (23).

At present our understanding of precise molecular and physiological roles of MetAP2 of insect microsporidia spp. is very limited. We therefore envision that the results of the present study could be utilized as a reasonable foundation, as we look ahead for the rational drug design, development of improved and highly specific anti-microsporidian agents, and field friendly diagnostic tools. The encouraging news is that MetAP2 is now seen as an extremely logical therapeutic target^12^ for microsporidia spp. For instance, it was reported that human microsporadian *Enterocytozoon bieneusi* (MetAP2c) and human MetAP2b structures have small amino acid differences in the active sites (S1 subsite). It was argued that these differences can be exploited to design fumagillin analogues that are specific for MetAP2 of microsporidian spp. (23). Although new inhibitors of this pathway hold great promise as therapeutic agents, drugs such as fumagillin and its derivatives were shown to have inhibitory effect on *MetAP2* of human microsporidia (70) and *Nosema ceranae* of honeybees (71).

Given that currently there is no established therapeutic agent to control “pebrine,” a deadly disease of silkworms caused by *Nosema bombycis*, it is important to utilize therapeutic agents like fumagillin and its analogues which are already tested on other microsporidia spp. This argument is reinforced by the fact that fumagillin still holds great promise in controlling Nosema infection in honeybees in the field conditions (72).

## Data availability statement

The datasets presented in this study can be found in online repositories. The names of the repository/repositories and accession number(s) can be found at: https://www.ncbi.nlm.nih.gov/genbank/, KX185053.1; https://www.ncbi.nlm.nih.gov/genbank/, PP576694.1.

## Ethics statement

The manuscript presents research on animals that do not require ethical approval for their study.

## Author contributions

IQ: Conceptualization, Data curation, Formal analysis, Investigation, Methodology, Software, Validation, Writing – original draft. TY: Data curation, Investigation, Methodology, Writing – review & editing. SY: Conceptualization, Data curation, Investigation, Methodology, Writing – review & editing. CA: Formal analysis, Software, Writing – review & editing. JL: Funding acquisition, Project administration, Resources, Supervision, Validation, Writing – review & editing.

## Footnotes

^1^ http://microsporidiadb.org/micro/

^2^ https://www.ncbi.nlm.nih.gov/orffinder/

^3^ https://blast.ncbi.nlm.nih.gov/Blast.cgi

^4^ https://www.ncbi.nlm.nih.gov/Structure/cdd/cdd.shtml

^5^ https://web.expasy.org/protparam/

^6^ https://dtu.biolib.com/DeepTMHMM

^7^ https://services.healthtech.dtu.dk/services/SignalP-6.0/

^8^ https://npsa-prabi.ibcp.fr

^9^ https://www.genscript.com/psort.html

^10^ https://services.healthtech.dtu.dk/services/DeepLoc-2.0/

^11^ https://swissmodel.expasy.org/

^12^ https://einsteinmed.edu/departments/pathology/research/microsporidia
