# Supplementary material for: Molecular characterization and phylogenetic analyses of MetAP2 gene and protein of Nosema bombycis isolated from Guangdong, China
Source: Front Vet Sci. 2024 Jun 28;11:1429169. doi: 10.3389/fvets.2024.1429169 (PMC11239577; doi:10.3389/fvets.2024.1429169)
Supplement: Supplementary file 1 [file Data_Sheet_1.docx]

**Molecular Characterization and Phylogenetic Analyses of MetAP2 gene and protein of *Nosema bombycis* isolated from Guangdong, China**

Izhar Hyder Qazi^1†^, Ting Yuan^1†^, Sijia Yang^1^, Christiana Angel^2^, Jiping Liu^1^*

^1^Guangdong Provincial Key Lab of Agro-Animal Genomics and Molecular Breeding, College of Animal Science, South China Agricultural University, Guangzhou 510642, Guangdong, China

^2^Shaheed Benazir Bhutto University of veterinary and Animal Sciences, Sakrand, 67210, Sindh, Pakistan.

^*^Corresponding author: Jiping Liu (e-mail: liujiping@scau.edu.cn)

^†^Equal contribution.

# Supplementary Material


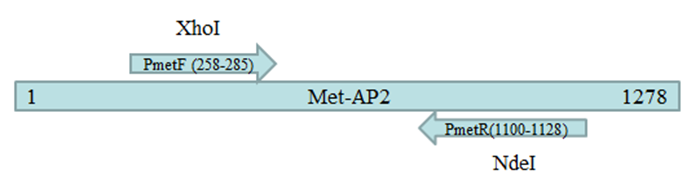


## Supplementary Figure 1: Strategy for Design of Primers for Prokaryotic Expression.


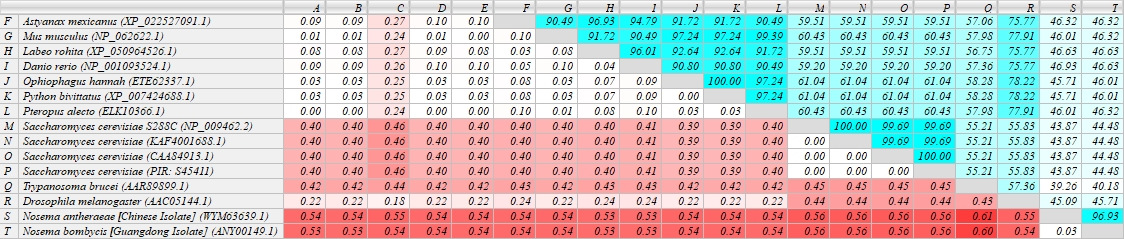


**Supplementary Figure 2:** The homology comparisons (%identity and divergence) between MetAP2 of *Nosema bombycis* (Guangdong isolate) and other spp.

Notes: Values in turquoise colored boxes denote the percent (%) identity, and values in red colored boxes denote the divergence. Color gradient highlights the values (%identity and divergence) from higher (dark) to lower (light) range. *MetAP2* genes of *Nosema bombycis* (Guangdong isolate) and *Nosema antheraeae* (Chinese isolate) were sequenced in the present study.


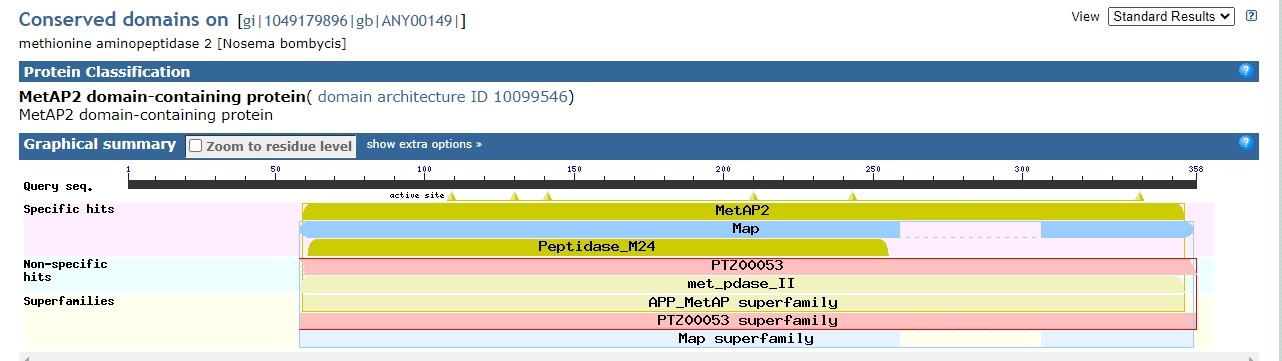


**Supplementary Figure 3** Domain prediction of *Nosema bombycis* (Guangdong isolate) MetAP2 protein.


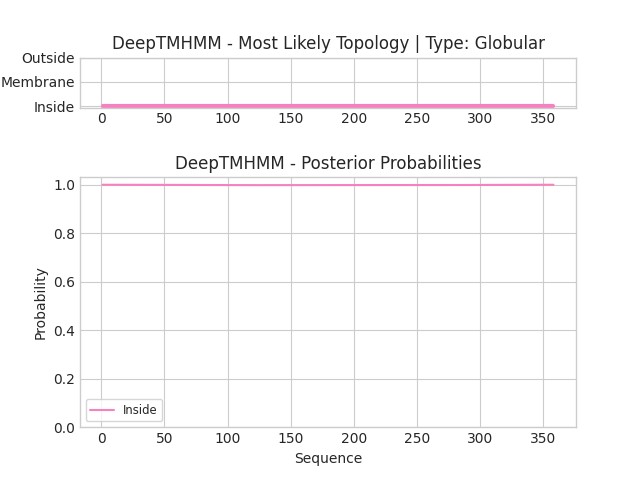


**Supplementary Figure 4:** Prediction of transmembrane domain structure of *Nosema bombycis* (Guangdong isolate) MetAP2 protein.


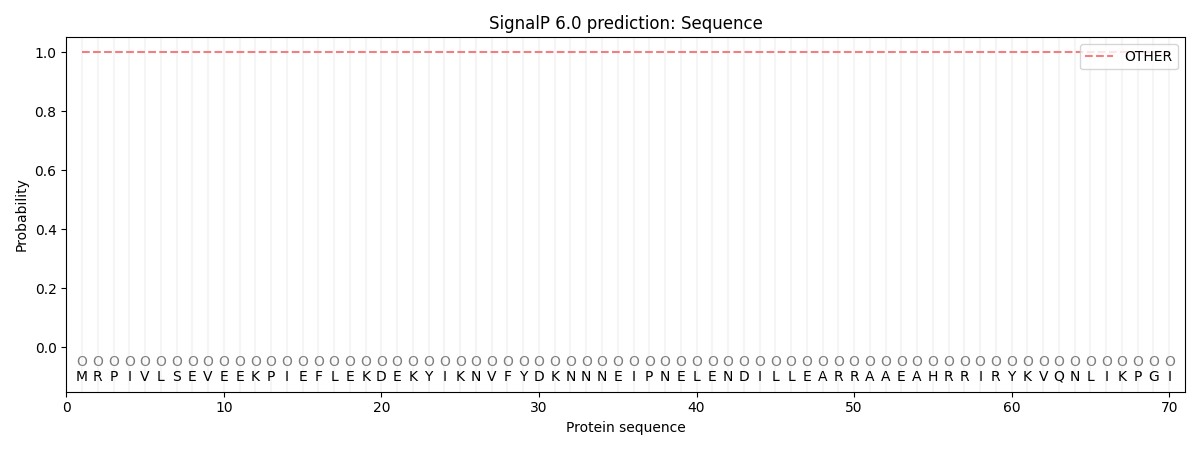


**Supplementary Figure 5** Prediction of signal peptide in *Nosema bombycis* (Guangdong isolate) MetAP2 protein.


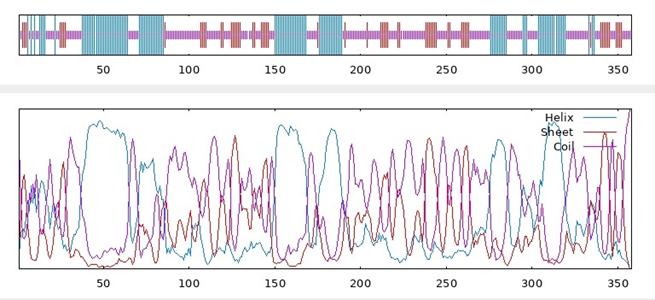


**Supplementary Figure 6** Secondary domain prediction of *Nosema bombycis* (Guangdong isolate) MetAP2 protein.

##
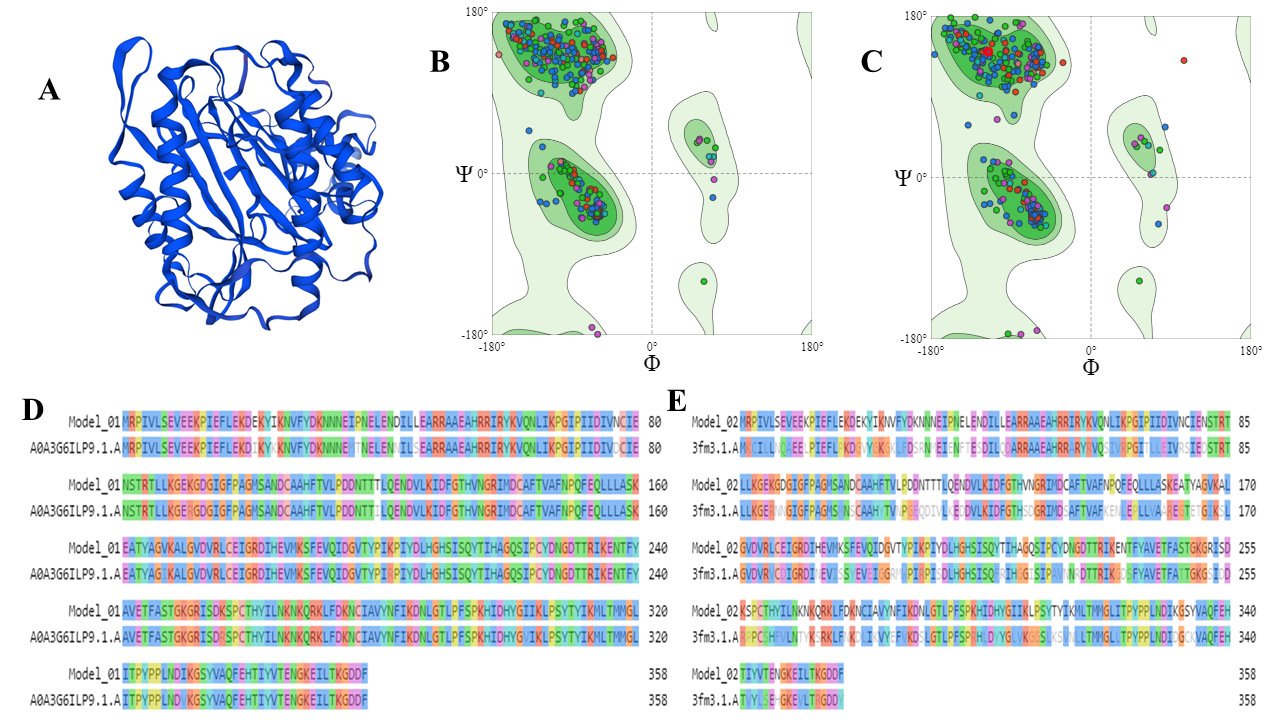


**Supplementary Figure 7** Homology Modeling of MetAP2 protein.

A) 3D structure of MetAP2 protein of *Nosema bombycis*. This model was developed based on template A0A3G6ILP9.1.A (MetAP2 *Nosema* *assamensis*). Color scheme of ribbon represents the confidence gradient (key shown in Figure 9A in the main text). B-C) The Ramachandran Favoured value for models 1 and 2, respectively. D-E) The target-template sequence alignments for models 1 and 2, respectively.


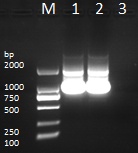


**Supplementary Figure 8** Primer MC-F/R agarose gel electrophoresis pattern.

Note: 1. *Nosema bombycis* DNA; 2. *Nosema bombycis* DNA; 3. Sterilized water; M: DL2000 Marker.


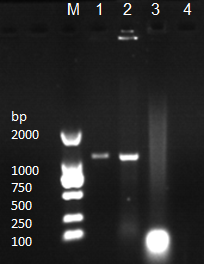


**Supplementary Figure 9** Identification results of bacterial liquid constructed with *MetAP2* gene cloning vector.

Note: 1. *Nosema bombycis* DNA; 2. Cloning vector bacterial solution; 3. Silkworm midgut; 4. Sterilized water; M: DL2000 Marker.


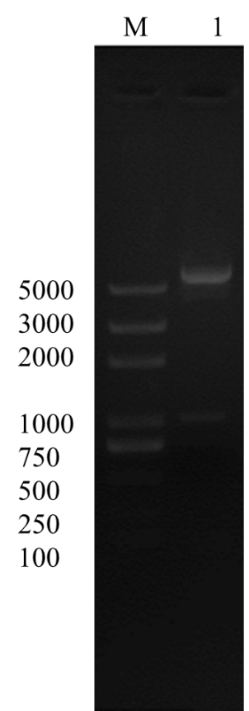

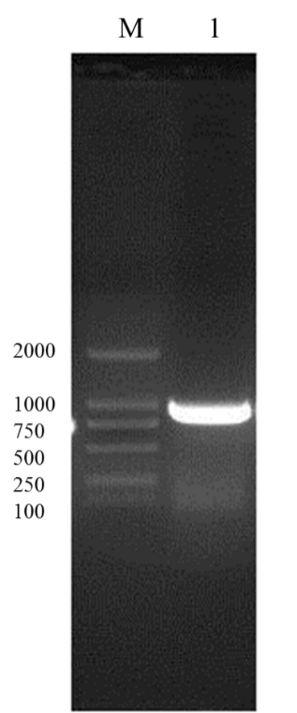


(B)

(B)

(A)

**Supplementary Figure 10** PCR identification and double enzyme digestion results of recombinant plasmid pET-28A–met.

Note: A represents the PCR identification result 1. *Nosema bombycis* DNA; M: DL2000 Marker; B represents the result of double enzyme digestion of the recombinant plasmid pET-28A-met. 1. NdeI and XhoI double enzyme digestion of pET-28A–met; M: DL5000 Marker.


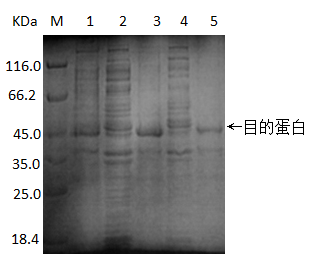


**Supplementary Figure 11** SDS-PAGE analysis of fusion proteins precipitated at different temperatures.

Note: 1. Total protein before induction; 2. 20℃ supernatant; 3. 20℃ precipitation; 4. 37℃ supernatant; 5. 37℃ precipitation; M: Protein Marker.

**
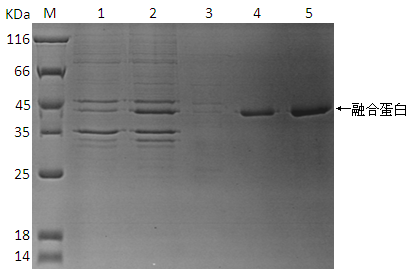
**

**Supplementary Figure 12** SDS-PAGE analysis of nickel agarose affinity chromatography purification**.**

Note: 1. Supernatant fraction; 2. Precipitate fraction; 3. 20 mM Imidazole elution fraction; 4. 50 mM Imidazole elution fraction; 5. 500 mM Imidazole elution fraction; M: Protein marker.
